# The Role of Non-Cognitive Factors in the SAT Remains Unclear: A Commentary on Hannon (2019)

**DOI:** 10.3390/jintelligence8020015

**Published:** 2020-04-13

**Authors:** Andrew R. A. Conway, Han Hao

**Affiliations:** Department of Psychology, Claremont Graduate University, Claremont, CA 91711, USA; han.hao@cgu.edu

**Keywords:** intelligence, cognitive ability, SAT

## Abstract

In the current issue of the *Journal of Intelligence*, Hannon (2019) reports a novel and intriguing pattern of results that could be interpreted as evidence that the SAT is biased against Hispanic students. Specifically, Hannon’s analyses suggest that non-cognitive factors, such as test anxiety, contribute to SAT performance and the impact of test anxiety on the SAT is stronger among Hispanic students than European-American students. Importantly, this pattern of results was observed after controlling for individual differences in cognitive abilities. We argue that there are multiple issues with Hannon’s investigation and interpretation. For instance, Hannon did not include an adequate number or variety of measures of cognitive ability. In addition, the measure of test anxiety was a retrospective self-report survey on evaluated anxiety rather than a direct measure of situational test anxiety associated with the SAT. Based on these and other observations, we conclude that Hannon’s current results do not provide sufficient evidence to suggest that non-cognitive factors play a significant role in the SAT or that they impact European-American and Hispanic students differently.

## 1. Introduction

In 2019, 3.7 million students graduated from high school in the United States. Approximately 60% of them, over 2.2 million students, took the SAT (National Center for Education Statistics). These students shared their SAT scores with colleges and universities across the country, to be included as part of their college applications. The majority of these institutions consider SAT scores to be of “moderate” to “considerably high” importance in college admissions decisions (Zwick 2012). Therefore, for high school students in the United States, the SAT is one of the most, if not the most, important standardized tests that they will ever take (Hannon 2012).

There is an ongoing debate about the use of standardized tests in education, especially with respect to the SAT and college admissions. Proponents of the SAT argue that the test provides an objective measure of cognitive ability and SAT scores can be used to predict college performance. Indeed, overall SAT scores are highly correlated with scores on standardized tests of intelligence (e.g., SAT and ASVAB *g*, r = 0.82 (Frey and Detterman 2004); SAT and ASVAB *g*, r = 0.73 (Coyle 2015), and SAT scores are correlated with college GPA (e.g., SAT and first-year GPA, r = 0.51 (Westrick et al. 2019); SAT and first-year GPA adjusted for course difficulty, r = 0.55 (Berry and Sackett 2008). Despite this overwhelming evidence for construct validity and predictive validity, the SAT is widely criticized. Some critics recognize the utility of the SAT but argue that test scores should be given less consideration in college admissions decisions. More extreme critics argue that the SAT (and standardized testing in general) is biased and should be abandoned.

In the current issue of the *Journal of Intelligence*, Hannon (2019) reports a novel and intriguing pattern of results that could be interpreted as evidence that the SAT is biased against Hispanic students. Specifically, Hannon’s analyses suggest that non-cognitive factors, such as test anxiety, contribute to SAT performance and the impact of test anxiety on the SAT is stronger among Hispanic students than European-American students. Importantly, this pattern of results was observed after controlling for individual differences in cognitive abilities. In other words, Hispanic and European-American students with the same level of cognitive ability may perform differently on the SAT due to test anxiety, and the difference will favor European-American students.

The group differences reported by Hannon could have serious implications for how we interpret SAT scores. However, Hannon’s results are inconsistent with previous findings with respect to the SAT. Firstly, as mentioned, overall SAT scores are highly correlated with the general factor (*g*) of the ASVAB (r = 0.82 and 0.73). This means that cognitive abilities explain between 50% and 75% of the variance in the SAT. In Hannon’s study, the aggregate of cognitive ability measures explained only 24% of the variance. It is therefore not clear if Hannon truly accounted for individual differences in cognitive ability when investigating the contribution of non-cognitive factors. Secondly, systematic investigations designed to detect test bias in the SAT and other standardized tests consistently fail to find evidence of bias (Linn 1973; Young 2001). For example, if the SAT is less reflective of cognitive ability in Hispanic students compared to European-American students then the correlation between SAT scores and college GPA should be lower in Hispanic students. Across multiple studies, however, this pattern of “underprediction” has not been observed (for a review, see Sackett et al. 2008).

We argue that there are multiple problems with Hannon’s investigation and interpretation, and we conclude that Hannon’s results do not provide sufficient evidence to suggest that non-cognitive factors play a significant role in the SAT or that they impact European-American and Hispanic students differently. Our conclusion is based on the following 4 observations:

### 1.1. Inadequate Measurement of Cognitive Abilities

Hannon reported a significant relationship between test anxiety and the SAT, which was moderated by ethnicity, *after controlling for cognitive abilities*. Controlling for cognitive abilities is necessary to make claims about the impact of non-cognitive factors on the SAT. For example, if cognitive and non-cognitive factors are correlated and cognitive factors are not fully accounted for, then the analysis will overestimate the impact of non-cognitive factors.

The problem with Hannon’s analysis is that “cognitive abilities” were measured with just two tasks: operation span and knowledge integration. This is an extremely limited approach to the assessment of cognitive ability. A more comprehensive approach would have included multiple constructs and multiple tasks per construct. For example, fluid reasoning and processing speed are two well established, broad cognitive abilities but were not assessed. In addition, working memory capacity is typically measured with multiple tasks, not just a single task. For example, Unsworth et al. (2012) administered three working memory tasks and found that the working memory factor was highly correlated with overall SAT scores, r = 0.66. This is substantially higher than the correlation between working memory and the SAT reported by Hannon (r = 0.38). Overall, Hannon reports that cognitive abilities account for 24% of the variance in the SAT. In contrast, Frey and Detterman (2004) found that cognitive abilities accounted for 67% of the variance in the SAT.

The implication of including just two measures is that Hannon did not adequately *control for cognitive abilities*. This makes it difficult to trust estimates of the impact of non-cognitive factors on the SAT. A more comprehensive measurement approach could improve the current investigation, for example, by using cognitive ability measures similar to those in Hannon’s previous work on the latent variable models of cognitive ability to identify sources of individual differences in reading comprehension (Hannon 2012).

### 1.2. Inappropriate Use of Causal Language

Test anxiety was measured with the Test Anxiety Scale (TAS), and the subjects in the study were college students. The SAT, however, was completed when the subjects were in high school. The TAS is, therefore, a retrospective self-report measure of anxiety rather than a measure of situational test anxiety specifically associated with the SAT. For comparison, consider the effect of stereotype threat on test performance. In a stereotype-threat experiment, a threat manipulation is introduced immediately prior to the administration of the test, and a group of participants that receive the threat is compared to a control group of participants that do not receive a threat. This allows for causal claims about the effect of threat on test performance. The correlation reported here between subjects’ current test anxiety and their SAT scores should be interpreted more conservatively than it was described in the study.

Hannon inappropriately invoked causal language in several places when interpreting the relationship between test anxiety and the SAT. For example, “…when Hispanic students’ test anxiety scores were high (e.g., a score of 33), test anxiety had a much larger negative influence on Hispanic students’ overall SAT scores.” This kind of statement implies that non-cognitive factors have a mechanistic role in the performance on the SAT, which is not a valid argument given the measure of test anxiety and the correlational nature of the data.

**Figure 1:**
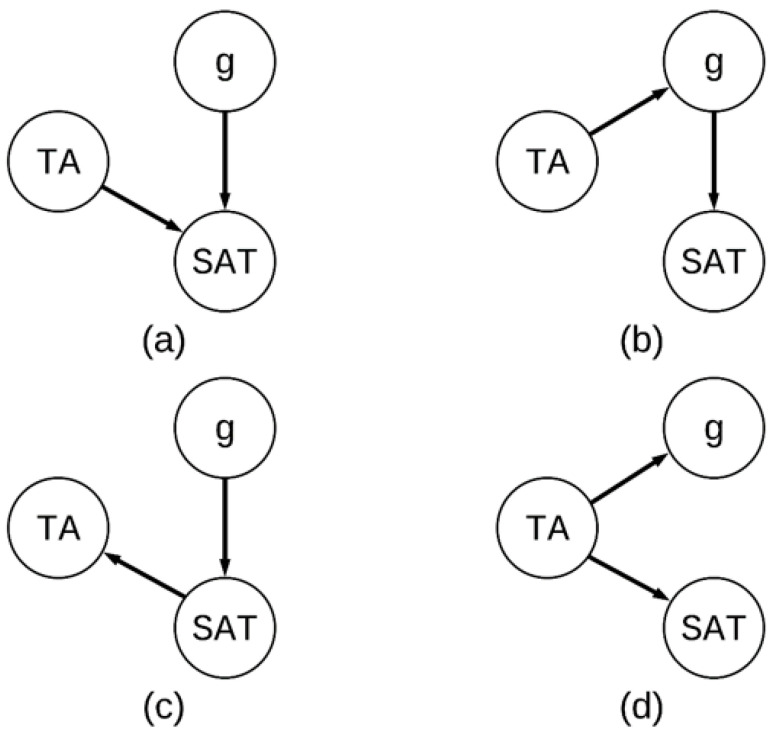
Four potential theoretical causal models among test anxiety (TA), general cognitive abilities (g), and SAT performance (SAT). (**a**) TA as a direct cause of SAT (**b**) TA as an indirect cause of SAT (**c**) SAT as a direct cause of TA; (**d**) TA as a direct cause of SAT and *g*.

In fact, there are several possible interpretations of the relationship between test anxiety, cognitive ability, and the SAT. Consider the four different models presented in Figure 1. Hannon’s interpretation is depicted in panel A; both test anxiety (TA) and cognitive ability (*g*) have an effect on the SAT. An interpretation consistent with the effect of stereotype threat is depicted in panel B; TA impacts *g,* which in turn impacts the SAT. However, it is also possible that TA does not have a direct effect on the SAT, which is depicted in panel C; *g* has an effect on the SAT, which causes TA. Finally, it is possible that TA has an impact on all assessments, which is depicted in panel D.

We realize that readers of the journal understand the difference between correlation and causation, but the point of this exercise is to illustrate that there were no predicted relationships between non-cognitive factors, cognitive abilities, and the SAT specified in Hannon (2019). Therefore, the exploratory analyses of correlational data in Hannon (2019) should be interpreted with caution.

### 1.3. Questionable Role of Test Anxiety

As mentioned, test anxiety was measured with the TAS, which was completed sometime *after* the subjects took the SAT. It is therefore not clear how to interpret the test anxiety score and its relationship to the SAT. Evidence suggests that the relationship between test anxiety and test performance varies depending on whether anxiety is measured before or after testing (Seipp 1991; Zeidner 1991). Specifically, the correlation between anxiety and performance is lower before testing than it is after testing, suggesting an effect of performance evaluation on anxiety in post-test self-report anxiety measures (Zeidner 1991). Thus, self-report test-anxiety measures completed *after* testing may be more dependent on test performance than self-report test-anxiety measures completed *before* testing, which is clearly a threat to construct validity. Furthermore, Hannon interprets the TAS score to reflect test anxiety as a trait, but it seems that the score could just as easily reflect attitudes toward tests or attribution of test performance to test anxiety. For example, a high score might indicate that the individual has an extremely negative attitude about tests or does not consider tests to be important.

It is also not clear why test anxiety would have a stronger negative impact on the SAT among Hispanic students compared to European-American students. The regression analyses included 10 non-cognitive predictor variables (five measures and the interaction terms) but there were no *specific* a priori predictions regarding the relationship between these variables and the SAT. In sum, this is an exploratory finding based on a retrospective self-report measure of test anxiety. In our view, this kind of exploratory result should be interpreted with extreme caution.

### 1.4. Misleading Plots

**Figure 2:**
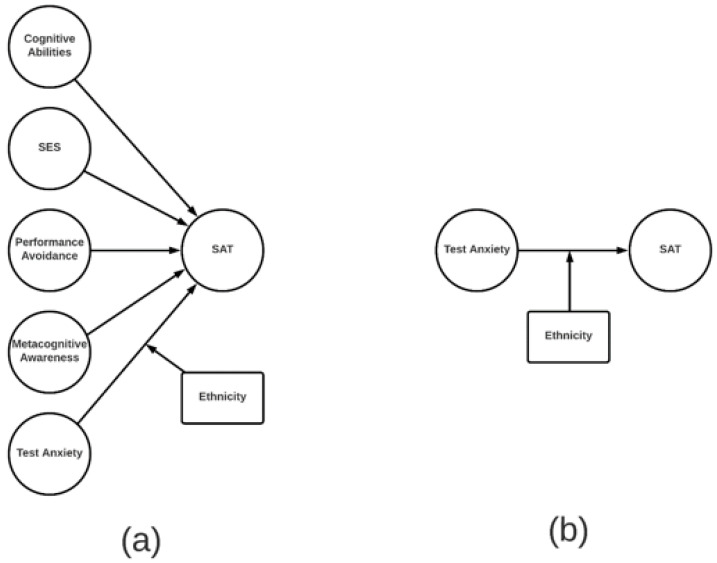
The theoretical difference between the regression results in Hannon (2019) Table 3 and the visualizations in Hannon (2019) Figures 1 and 2. Left panel (**a**) demonstrates the regression results and right panel (**b**) demonstrates what was plotted in Figures 1 and 2. SES refers to Socioeconomic Status.

Finally, Hannon exaggerates the magnitude of group differences in the plots displayed in Figure 1 and Figure 2. The predicted SAT scores plotted in the figures are inappropriately based on the interaction term of ethnicity and test anxiety only, while the true regression coefficients/intercepts are from a multiple regression model with other cognitive and non-cognitive predictors (see Figure 2 for an illustration of the difference). Even after controlling for all other factors without significant interactions with ethnicity, the predicted scores should be based on the main effects of test anxiety and ethnicity and their interaction.

**Figure 3:**
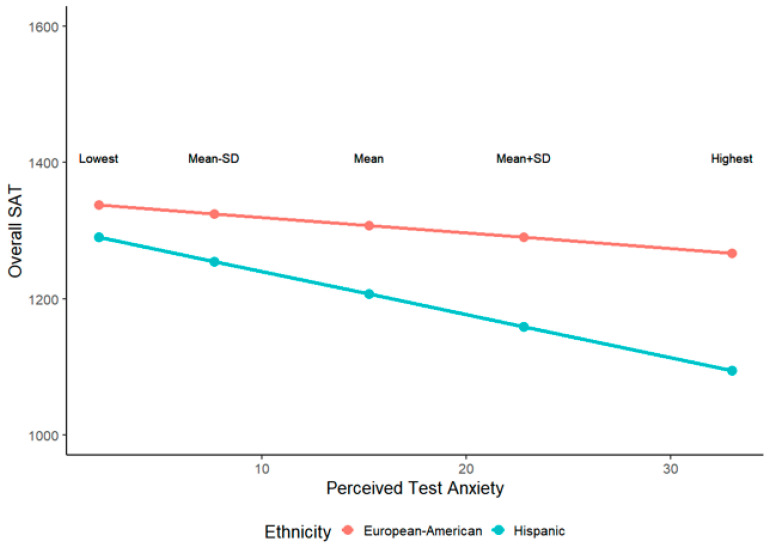
Reproduced visualization of overall SAT scores as a function of ethnicity and test anxiety, and their interaction when controlling for cognitive abilities, performance-avoidance, metacognitive awareness, and SES.

**Figure 4:**
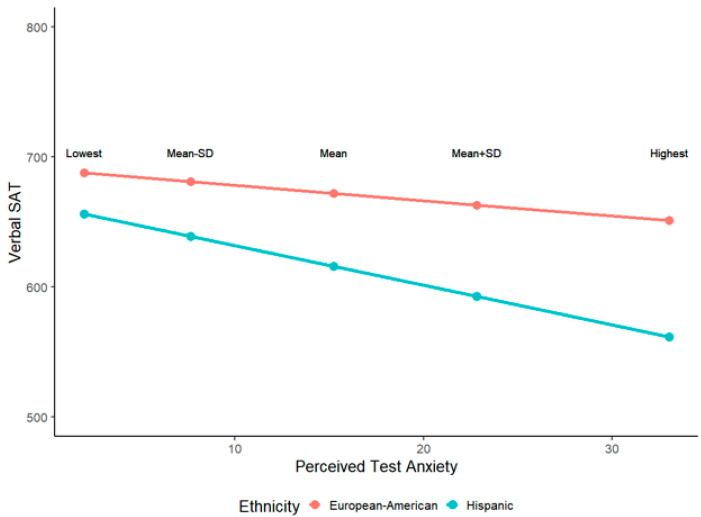
Reproduced visualization of verbal SAT scores as a function of ethnicity and test anxiety, and their interaction when controlling for cognitive abilities, performance-avoidance, metacognitive awareness, and SES.

More importantly, Hannon plotted predicted SAT scores based on extreme test-anxiety scores (minimum and maximum). A more conventional approach is to plot predicted scores on Y based on the mean of X, the mean of X plus 1 standard deviation, and the mean of X minus 1 standard deviation. Plots of these predicted SAT scores are presented in Figure 3 and Figure 4.

Finally, a more comprehensive approach to the analysis of group differences would be to use structural equation modeling (SEM) and test for measurement and structural invariance across groups (e.g., a multi-group SEM analysis of the full structural regression model depicted in Figure 2). This approach may be particularly beneficial when investigating potential biased predictors. Specifically, a test for structural invariance can help to determine whether regression coefficients for direct effects vary across groups (Kline 2015). Potential ethnicity differences in the overall relationship between cognitive and non-cognitive factors and the SAT would be reflected in a comparison of the multi-group structural model with all latent path coefficients freely estimated and a model with all latent paths fixed across ethnicity groups. Potential ethnicity differences on the specific relationship between test anxiety and the SAT would be reflected in a comparison of the multi-group model with the regression path from test anxiety to the SAT being freely estimated across ethnicity groups and a model with that path being fixed across groups.

Based on the above four considerations, we argue that there is insufficient evidence to conclude that non-cognitive factors play an important role in the SAT or that non-cognitive factors exert a greater influence on Hispanic students than European-American students.

## 2. What Does the SAT Measure?

The question remains, what does the SAT measure? In our view, the SAT resembles any other battery of tests designed to measure intelligence. That is, the overall test is designed to measure a few broad abilities, and those broad abilities are positively correlated. In other words, the VSAT is a measure of verbal ability, the QSAT is a measure of mathematical ability, and *g* represents the covariance between VSAT and QSAT. The question is, how do we interpret *g*? Is it a general mental ability or is it an index of overall cognitive ability?

According to the process overlap theory (Kovacs and Conway 2016; Kovacs and Conway 2019), *g* is simply an index of overall cognitive ability. In other words, *g* should not be interpreted as a psychological attribute. According to the process overlap theory (POT), the covariance among broad cognitive abilities (*g*) is observed because the cognitive processes that are required to perform test items are sampled in an overlapping manner across a battery of tests. This “process overlap” results in a general factor despite the fact that there is no general ability. That said, the POT does assume that domain-general processes, which are associated with executive attention and working memory, are sampled more often than domain-specific processes, and so they contribute more to a measure of *g*.

With respect to the SAT, this means that the test measures verbal ability and mathematical ability (broadly defined) and domain-general processes associated with attention and working memory. While it may be true that the SAT measures some non-cognitive factors, we remain skeptical about the importance of such factors; how much variance do they actually account for (above and beyond cognitive ability), and is their contribution stable across tests and testing environments?

## 3. Conclusions

In conclusion, Hannon (2019) reported a pattern of novel and potentially important results with respect to group differences in the SAT. However, upon further investigation of the study, we argue that there is not sufficient evidence in the current study to warrant the claim that the SAT measures non-cognitive factors, or that non-cognitive factors contribute more to the SAT for any one group of students relative to another. That said, previous work does suggest that non-cognitive factors play a small but significant role in the SAT (Hannon 2012). However, the majority of the variance in the SAT can be explained by a combination of domain-general (attention and working memory) and domain-specific (verbal and math) cognitive abilities.
